# Association between parities and duration of breastfeeding and the severity of coronary artery disease in women above 30 years old age (A pilot study) 

**DOI:** 10.22088/cjim.15.3.430

**Published:** 2024-08-01

**Authors:** Mirhossein Seyyed-mohammadzad, Dorsa Kavandi, Mohammad Jalili, Sahar Ghodratizadeh, Amir Mikaeilvand, Hanieh Sakha, Reza Hajizadeh

**Affiliations:** 1 Department of Cardiology, Urmia University of Medical Sciences, Urmia, Iran; 2 Student Research Committee, Zanjan University of Medical Sciences, Urmia, Iran; 3 Department of Anesthesiology, Urmia University of Medical Sciences, Urmia, Iran; 4 Cardiovascular Research Center, Tabriz University of Medical Sciences, Tabriz, Iran

**Keywords:** Breastfeeding, Livebirths, Syntax score, Coronary artery disease, Pregnancy

## Abstract

**Background:**

The prevalence and mortality of CVD in women increase over time. We conducted this research to evaluate the severity of coronary artery disease with the number of live births and breastfeeding duration.

**Methods:**

Patients aged 30-50 years old with positive exercise tests or evidence of cardiac ischemia who were candidates for coronary angiography were included. All the participants had at least one child. Syntax score was used to evaluate the severity of coronary arteries.

**Results:**

Mean number of children was 3.72±1.85, in those patients with <2 live births no one had a syntax score≥1, but in the>5 live births group most patients had a syntax score≥1. In patients with zero syntax score, it was estimated as 4.91±39.7; in patients with 1≤ syntax score, it was 4.48±7.29 (P =0.76). Among patients with > 5 birth lives, those with higher syntax scores had older ages (P=0.497). After adjusting age, the association between live births and syntax score became non-significant (P=0.850).

**Conclusion:**

By increasing the number of live births >5, the severity of coronary artery disease, increases. However, this association was not significant after adjusting the age of patients.

Cardiovascular disease (CVD) is a common cause of death worldwide (1, 2). At younger ages, men are more prone to CVD than women, but the lifelong risk is determined almost equally in both genders (3). The prevalence and mortality of CVD in women increase over time, especially after the menopause transition (2). CVD mortality is reported in one in three women throughout the world (4). 

 In addition to all known risk factors of CVD such as hypertension, diabetes mellitus, hyperlipidemia, obesity, and smoking which have similar effects in both genders, female-specific risk factors play an important role in the prediction of coronary heart disease (CHD) in women’s later life (5-7). It is proven that early onset of menstrual period (menarche), menopause at a younger age, hysterectomy, and physiologic changes in pregnancy is related to a higher CVD risk (8, 9). Women experience hormonal changes throughout their lives that can result in metabolic adverse effects on the cardiovascular system. Increasing lipid levels, insulin resistance, and central fat accumulation are among those changes during pregnancy and after menopause transition (10). Estrogen has a protective effect in women protecting them against cardiovascular diseases including cerebrovascular disease and ischemic heart disease (11). 

It has been shown that estrogen deficiency is associated with dyslipidemia and increased body cholesterol and LDL-c in menopausal women, on the other hand, follicle-stimulating hormone (FSH) could be the cause of high cholesterol production in the liver (12-14). One study showed that improvement in lipid profile was achieved by 30% lowering FSH concentrations (15). During pregnancy, FSH is decreased, but its levels gradually increase after delivery. During one year after birth, FSH level is in the upper normal range returning to the normal range after one year (16). All these findings show that the interaction between pregnancy, breastfeeding, and hormonal changes and their effect on the cardiovascular system is sophisticated and multifactorial. 

According to the 2011 American Heart Association guidelines, obstetrical unfavorable events should be considered in the evaluation of CVD risk in middle-aged or older women (17). Reproductive history including the number of parities and duration of breastfeeding has attracted the most attention in recent studies. Although physiological changes in pregnancy are essential for both the fetus’s growth and preparing the mother for breastfeeding, these changes have short and long-term effects on the maternal cardio-metabolic profile (18). On the other hand, breastfeeding is believed to reverse these negative metabolic effects of pregnancy (7, 13) and help prevent metabolic syndrome and CVD risk (7, 19). The fact that the longer the duration of lactation is related to the lower the risk of CVD could reveal this protective effect (7, 20). Few studies have evaluated the association between the number of live births and CVD occurrence. Some articles have demonstrated that higher rates of parities are associated with a greater risk of cardiovascular diseases (17, 20). While other studies observed no association between parities and CVD risk and mortality (21, 22). On the other hand, some researchers showed a U-shaped (J-shaped) relationship between number of parities and CVD risk/mortality (1, 17, 23). On both ends of the spectrum, nulliparous and multiparous women are at high risk for CVD (1, 17), but a longer duration of breastfeeding might modulate the negative effect of higher parities. To the best of our knowledge, there is no study evaluating the relationship between parities and breastfeeding with the severity of each coronary artery involvement. In this study, we evaluated the association between the severity of coronary artery stenosis evaluated by Syntax score and the number of parities and, duration of breastfeeding. We hope that this research could give a better view of interactions between pregnancy and breastfeeding and their effect on the cardiovascular system and help health providers to improve future guidelines.

## Methods

**Study Design and Oversight:** From September 2020 to September 2021, this cross-sectional study was done in a tertiary center, after getting an approval license from the university's ethical committee. The study plan was designed as a pilot study including 108 patients. After meeting (matching) the inclusion and exclusion criteria finally 104 patients, participated in this study. They were asked about the number of deliveries, duration of breastfeeding, and type of delivery. Regarding previous studies; participants were placed into the groups as follows (17):

- **Number of deliveries** (live birth):

1. No live birth (Zero),

 2. One or two live births, 

 3. Between 3 to 5 live births,

 4. Five or more live births.

- **Duration of lactation**:

1. Below (less than) 2 months, 

2. Between 2 to 12 months. 

3. Over (more than) 12 months.

- **Type of deliveries**: 1. Cesarean section, 2. Vaginal delivery

The above data were recorded using an interviewer-administered questionnaire. All patients underwent coronary angiography according to standard protocols and the severity of coronary artery stenosis was assessed using syntax score. Demographic data and other information including past medical history, past reproductive history, smoking, and family history of cardiovascular disease of all participants were recorded. Patients were reassured that their information would remain confidential and all participants gave a written informed consent.

**Study population:** Patients were premenopausal Asian women aged 30-50 years old with positive exercise test or evidence of cardiac ischemia who were candidates for coronary angiography. All the participants had at least one child. Patients with prior CVD, stroke or cancer, and missing data on pregnancies/births, or lactation were excluded from this study.

**Percutaneous coronary intervention:** The coronary angiography was performed using the standard Judkins technique. Under cardiac monitoring and pulse-oximetry and after local anesthesia with 10-15 cc lidocaine, a small cut was made in the skin near the groin. The catheter was inserted into the femoral artery and was carefully guided to the coronary arteries. Then contrast agent was injected through the catheter and series of X-ray were taken when the contrast flowed through the blood vessel. Then, angiography recordings were evaluated by two expert cardiologists.

**Clinical outcomes and **statistical analyses**:** The primary end point of this study was to evaluate the effect of parities and duration of breastfeeding on the severity of coronary artery disease in women above 30 years old. The severity of CAD was measured by syntax score.

**Syntax score:** Syntax score is an anatomical scoring system that is clinically useful for stratifying CAD (clinical outcomes/prognosis) and making suitable treatment plans (revascularization modality, reperfusion method). The Syntax score was calculated by two expert cardiologists. Each coronary lesion with luminal narrowing ≥50% in vessels ≥1.5 mm in diameter was scored using the SYNTAX score calculator (available online at http://www.syntaxscore.org). Recent studies have demonstrated that syntax score is significantly associated with prognosis following PCI. Patients with high syntax score will have the worst prognosis (24).

## Results

Among the 104 women included in this study, the mean age of patients was 50.36±5.86 years. Table 1 shows patients’ demographic and obstetric data according to their Syntax score. The number of live births for each patient was recorded. Minimum and maximum number of live births was one and ten offspring, respectively. Mean number of children was 3.72±1.85. Table 2 shows the association between number of live births and the Syntax score. As table 2 shows, in <5 live births group most patients had a syntax score=zero, but in >5 live births group most patients had a syntax score≥1. The mean marriage age was 18.64± 4.33 years. 

**Figure 1:**
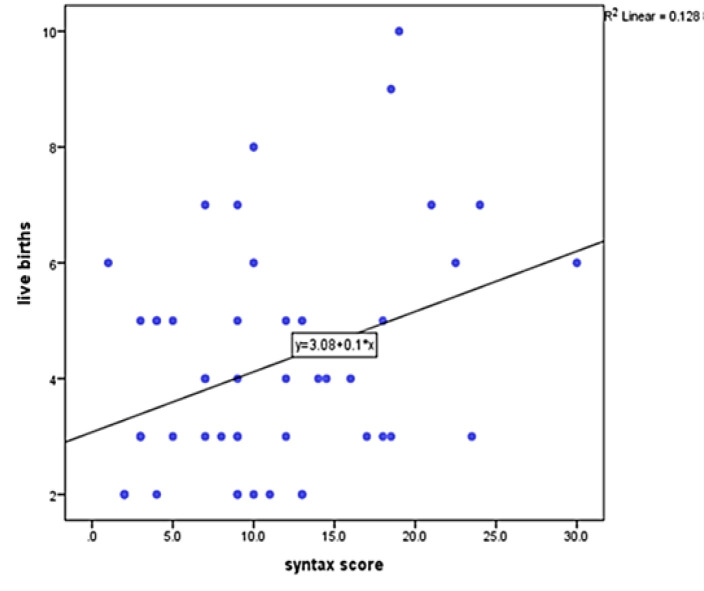
The association between Syntax score and live births

The minimum and maximum durations of lactation were zero and 336 months, respectively. Mean duration was calculated as 62.45±51.14 months. Figure 1 shows the association between Syntax score and live births. Association between mean duration of breastfeeding (months) and Syntax score was measured. Mean duration of breastfeeding in patients with zero syntax score was 57.33±40.35 months, and in patients with 1≤ syntax score was reported as 68.2±60.96 months. (p>0.05) Table 3 shows the association between breastfeeding duration (months) and Syntax score.

**Table 1:** Patients’ demographic and obstetric data according to their Syntax score

**Characteristics**	**Total**		**Syntax score=0**	**Syntax score≥1**	**P- value**
**Age at study entry, years Mean (SD)**	50.36±5.86		49.67±5.82	51.12± 5.87	_
**Weight Mean (SD)**	77.25± 10.95		79.13±11.34	75.14±10.20	_
**Diabetes (%)**	Positive:	46 (44.2%)	18 (32.7%)	28 (57.1%)	0.12
	Negative:	58 (55.8%)	37 (67.3%)	21 (42.9%)	
**Hypertension (%)**	Positive:	63 (60.6%)	32 (58.2%)	31 (63.3%)	0.59
	Negative:	41 (39.4%)	23 (41.8%)	18 (36.7%)	
**Hyperlipidemia (%)**	Positive:	25 (24%)	15 (27.3%)	10 (20.4%)	0.41
	Negative:	79 (76%)	40 (72.7%)	39 (79.6%)	
**Family history of IHD**	Positive:	58 (55.8%)	35 (63.6%)	23 (46.9%)	0.08
	Negative:	46 (44.2%)	20 (36.4%)	26 (53.1%)	
**Smoking**	Positive:	4 (3.8%)	3 (5.5%)	1 (2%)	0.366
	Negative:	100 (96.2%)	52 (94.5%)	48 (98%)	
**Hypertensive disorders in pregnancy**	Positive:	10 (9.6%)	7 (12.7%)	3 (6.1%)	0.25
	Negative:	94 (90.4%)	48 (87.3%)	46 (93.9%)	
**Gestational diabetes mellitus**	Positive:	13 (12.5%)	5 (9.1%)	8 (16.3%)	0.26
	Negative:	91 (87.5%)	50 (90.9%)	41 (83.7%)	
**Macrosomia (4 kg<infant weight)**	Zero:	81 (77.9%)	43 (78.2%)	38 (77.6%)	0.8
	One:	22 (21.2%)	12 (21.8%)	10 (20.4%)	
	Two:	1 (0.9%)	0	1 (2.0%)	
**Number of abortions (%)**	Zero:	71 (68.3%)	38 (69.1%)	33 (67.3%)	0.63
	One:	24 (23.1%)	14 (25.5%)	10 (20.4%)	
	Two:	5 (4.8%)	2 (3.6%)	3 (6.3%)	
	Three:	2 (1.9)	1 (1.8%)	1 (1.8%)	
	Four:	2 (1.9%)	0	2 (4.1%)	
**Still births (%)**	Zero:	97 (93.3%)	52 (94.5%)	45 (91.8%)	0.35
	One:	5 (4.8%)	3 (5.5%)	2 (4.1%)	
	Two:	2 (1.9%)	0	2 (4.1%)	
**Cesarean section**	Positive	35 (33.7%)	22 (40%)	13 (26.5%)	0.147
	Negative	69 (66.3%)	33 (60%)	36 (73.5%)	

**Table 2:** The association between number of live births and Syntax score

**Number of live births**	**Total**	**Syntax score**		**P- value**
		**0**	**≥1**	
**≤5**	89	51(92.7%)	38(77.6%)	0.002
**>5**	15	4(7.3%)	11(22.4%)	
**total**	104	55(100%)	49(100%)	

**Table 3:** The association between breastfeeding duration (months) and Syntax score

**breastfeeding duration**	**total**	**Syntax score**		**P-value**
		**0**	**1≤**	
**<2 months**	52(51.5%)	29(53.7)	23(48.9%)	0.81
**2-12 months**	25(24.8%)	12(22.2)	13(27.7%)	
**2months<**	24(23.8%)	13(24.1)	11(23.4%)	

**Coronary artery involvements:** Tables 4 and 5 show the association between live births and breastfeeding duration and coronary artery involvement. LAD and LCX vessels had a strong association with high live births, but there was no association between breastfeeding duration and coronary artery involvement.

The association between syntax score and breastfeeding duration divided into number of live parities also was evaluated. In patients with zero syntax score,it was estimated as 4.91±39.7 and in patients with 1≤ syntax score, it was 4.48±7.29 (P =0.76). Because age is an important factor, we adjusted patients according to their age. The mean age of patients with less than 5 live births and > 5 birth lives were 48.96±6.33 and 53.65±2.38 respectively (p<0.001). Among the patients with > 5 birth lives, those with higher syntax scores had older ages (P=0.497). After adjusting age, the association between live births and syntax score became non-significant (P=0.850).

**Table 4:** Association between live births and left anterior descending (LAD) coronary artery involvement

**Variables**		**Total**	**Number of live births**		**P-value**
			**5<**	**≥5**	
**LAD involvement**	0	39(37.5%)	29(39.7%)	10(32.3%)	0.04
	<50%	38(36.5%)	30(41.1%)	8(25.8%)	
	50%<	27(26%)	14(19.2%)	13(41.9%)	
**LCX involvement**	0	72(69.2%)	56(76.7%)	16(51.6%)	0.04
	<50%	21(20.2%)	11(15.1%)	10(32.3%)	
	50%<	11(10.6%)	6(8.2%)	5(16.1%)	
**RCA involvement**	0	64(62.7%)	48(67.7%)	16(51.6%)	0.25
	<50%	20(19.6%)	13(18.3%)	7(22.6%)	
	50%<	18(17.6%)	10(14.1%)	8(25.8%)	
	Total	104(100%)	71(100%)	31(100%)	

LAD; left anterior descending, LCX; left circumflex, RCA; right coronary artery

**Table 5:** The association between breastfeeding duration and coronary artery involvement

**Varibles**		**Number of patient**	**Mean(months of lactation)**	**SD**	**P- value**
**LAD angiography**	normal	39	59.92	40.96	0.9
	<50%	38	61.84	51.64	
	50%≤	27	66.96	63.89	
**LCX angiography**	normal	72	58.86	50.92	0.41
	<50%	21	71.81	58.192	
	50%≤	11	68.09	37.956	
**RCA angiography**	normal	64	58.39	43.53	0.67
	<50%	20	58.5	35.277	
	50%≤	18	82.06	83.11	

LAD; left anterior descending, LCX; left circumflex, RCA; right coronary artery

**Figure 2:**
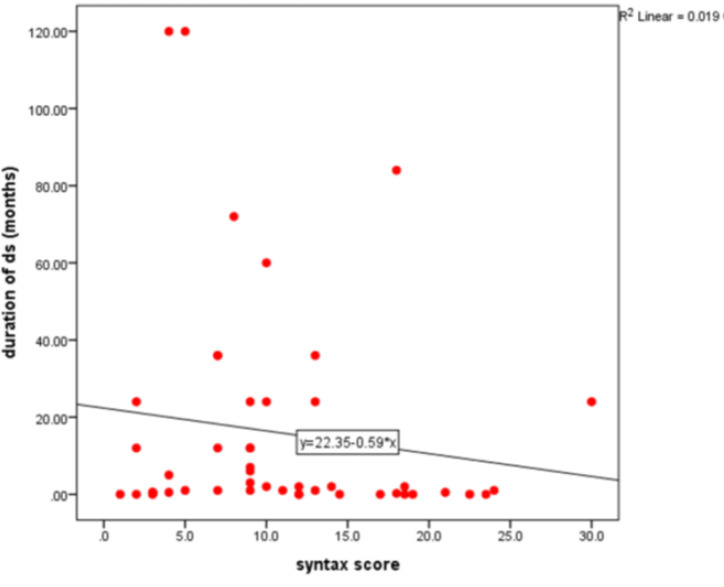
The Association between breast feeding duration and syntax score

## Discussion

According to the best of our knowledge, this is the first study that assessed the relationship between parities and breastfeeding with the severity of coronary artery disease. In this investigation, the number of parities had a significant association with the severity of coronary artery disease assessed by syntax score, but after entering age as a main risk factor for coronary artery disease, the association became non-significant.

Many studies have evaluated the role of reproductive dependent variables (gravity, parity, delivery type, lactation duration, mother’s age at first delivery and etc.) in prediction of lifetime CVD risk and mortality in women (20, 25) and reported conflicting outcomes. Some articles showed a U-shaped association between number of live births and CVD risk/mortality which indicate that nulliparous and multiparous women (both ends of the spectrum) are at high risk for CVD (17, 23). However, some other studies demonstrated a direct linear relationship between these parameters (6, 20). 

Lijun Shen et al. conducted a study to investigate whether parity affects the CHD occurrence in middle-aged and older Chinese women or not. Their results revealed that women with two or more children, were at increased risk for CHD and they proposed multi-parity as a risk factor for CHD (6). Also, in a multi-ethnic community-based cohort study by Ogunmoroti et al. multi-parity was significantly associated with reduced cardiovascular health (4).

According to Japanese cohort study even in a U-shaped association model, multiparous women were more prone to CVD mortality than nulliparous women (1). Nulliparous women were likely to have some disorders or risk factors leading to infertility, risks such as; polycystic ovarian syndrome (PCOS) and unhealthy manner of living or habits like smoking and drinking (23). These factors independently increase CVD risk. In addition, Chang et al. revealed that social communication may reduce the stress level and help to prevent neuroendocrine response and sympathetic nervous system stimulation, consequently reversing the pathogenesis of CHD (26). They indicated that nulliparous women were less interested to have husband and therefore had less social communication, which makes them susceptible to CVD (26). Furthermore, Parikh et al. attributed the increased risk of CVD in women with 0-1 live births to PCOS, obesity and thyroid disorders (27). 

In contrast to mentioned studies, some researchers found no considerable relationship between number of parities and cardiovascular events (21, 22). Additionally, some others have observed inverse relationship (25). Jacobs et al. performed a prospective cohort study and showed lower risk of CVD and CVD (except CHD) mortality in multi-gravid (four ≤ pregnancies) women (25). 

Gravidity (number of pregnancies) and parity (number of live births) both observed to have relationship with cardiovascular risk factors (25). Therefore, women who experience pregnancy loss (miscarriage, stillbirth or medical abortion) are at greater risk for CHD according to previous studies (17). Our survey showed that women with history of 0-1 miscarriage, were in zero syntax score group. However, women with 2-4 miscarriages had 1≤ syntax score. Furthermore, number of stillbirths in syntax≥1 group was higher than than zero syntax score group. This relationship between pregnancy loss with increased CHD risk is explained by underlying causes of miscarriage including immunologic disorders, endothelial dysfunction, and chronic diseases (17, 28). 

Several mechanisms have been detected to describe how CVD risk increases in multiparous women. Physiologic, hormonal, and metabolic changes during pregnancy, affect the lifelong cardio-metabolic profile. These changes include increased insulin resistance, glucose intolerance, rising serum lipid levels (LDL, triglycerides and total cholesterol), weight gain (increasing BMI), accumulation of abdominal (visceral) fat and reduced estrogen level (4, 17, 20). These factors have interaction with known cardiovascular risk factors (25). Majority of studies indicated that these adverse changes could be inverted by lactation in the postpartum period (29). Lactation influences lipid metabolism and lowers the risk of metabolic syndrome (30). Also, it can help to return to normal (pre-pregnant state) weight faster (soon) after delivery. A systematic review and meta-analysis study evaluated the relationship between duration of breastfeeding and the risk of metabolic syndrome. Researchers concluded that women with longer breastfeeding duration had lower serum levels of glucose, triglyceride and cholesterol (19). 

Although breastfeeding could have beneficial effects for both mother and baby, the duration of breastfeeding is also important. Longer duration of breastfeeding has more protective effect (30, 31). Lactation for more than 24 months during total life showed to have beneficial effects on cardiovascular health (30). Rajaei et al. reported that among parous women, those who breastfed months≥5 had lower risk for CAD later in life than those who did not breastfeed or breastfed less than 5 months (7). Our study showed that women with high syntax score had history of shorter breastfeeding duration in comparison to parous women with zero syntax score. Previously, Coldis et al. in 1987, showed that nulliparous women were at higher risk for cardiovascular diseases, while the number of live births did not affect cardiovascular risk (32). It is possible that cardiovascular events have increased in women with higher parities during past decades. Lauria et al. showed that only 34% of children who were at 12 months old had exclusive breastfeeding (33). New and qualified artificial milk products, and difficulties for breastfeeding among mothers who start their job soon after the delivery, are some possible causes of decreasing the incidence of breastfeeding. Most studies show that the duration of breastfeeding is not acceptable in most communities (34, 35), in developing countries, the trend for breastfeeding in urban regions is worse than rural regions (36).

Relationship between each coronary artery involvement and breastfeeding duration was also assessed. There was not significant association between breast feeding duration and selected coronary artery involvement.

The most accepted theory explained by the Peters et al.’s case– cohort study. They revealed that parities increase the risk of CHD, however breastfeeding declines this risk. Therefore, they concluded that parity is more potent factor than breastfeeding in determination of CHD risk (20). In addition, another study confirmed this result and indicated that parity could neutralize the positive effects of breastfeeding especially after four delivery (30). The age in an important risk for ischemic heart disease which could be missed in some investigations. Our outcomes confirm that the severity of the coronary artery involvement was strongly associated with number of live births rather than duration of breastfeeding. After adjusting our results with age of patients the association between number of parities and the severity of coronary artery disease became non-significant. With more studies and including larger sample size, we could find the effect of breastfeeding more accurately.

In conclusion, our study showed that by increasing the number of live births >5 live births, (possibly concomitant with decreased breastfeeding duration), the severity of coronary artery involvement increases. However, this association was not significant after adjusting the age of patients. Further studies are required to evaluate the interactions between multi-parity, breastfeeding duration and cardiovascular system.
